# Resequencing and annotation of the *Nostoc punctiforme* ATTC 29133 genome: facilitating biofuel and high-value chemical production

**DOI:** 10.1186/s13568-017-0338-9

**Published:** 2017-02-16

**Authors:** Luis E. Moraes, Matthew J. Blow, Erik R. Hawley, Hailan Piao, Rita Kuo, Jennifer Chiniquy, Nicole Shapiro, Tanja Woyke, James G. Fadel, Matthias Hess

**Affiliations:** 1 Department of Animal Science, University of California, Davis, 2251 Meyer Hall, Davis, CA 95616 USA; 2 Department of Energy, Joint Genome Institute, Walnut Creek, CA 94598 USA; 3 ZeaChem, Boardman, OR 97818 USA; 4 Washington State University, Richland, WA 99354 USA

**Keywords:** *Nostoc punctiforme*, Cyanobacteria, Carbon cycle, Nitrogen cycle, Natural product synthesis, Single molecule real-time sequencing

## Abstract

Cyanobacteria have the potential to produce bulk and fine chemicals and members belonging to *Nostoc* sp. have received particular attention due to their relatively fast growth rate and the relative ease with which they can be harvested. *Nostoc punctiforme* is an aerobic, motile, Gram-negative, filamentous cyanobacterium that has been studied intensively to enhance our understanding of microbial carbon and nitrogen fixation. The genome of the type strain *N. punctiforme* ATCC 29133 was sequenced in 2001 and the scientific community has used these genome data extensively since then. Advances in bioinformatics tools for sequence annotation and the importance of this organism prompted us to resequence and reanalyze its genome and to make both, the initial and improved annotation, available to the scientific community. The new draft genome has a total size of 9.1 Mbp and consists of 65 contiguous pieces of DNA with a GC content of 41.38% and 7664 protein-coding genes. Furthermore, the resequenced genome is slightly (5152 bp) larger and contains 987 more genes with functional prediction when compared to the previously published version. We deposited the annotation of both genomes in the Department of Energy’s IMG database to facilitate easy genome exploration by the scientific community without the need of in-depth bioinformatics skills. We expect that an facilitated access and ability to search the *N. punctiforme* ATCC 29133 for genes of interest will significantly facilitate metabolic engineering and genome prospecting efforts and ultimately the synthesis of biofuels and natural products from this keystone organism and closely related cyanobacteria.

## Introduction

Cyanobacteria are capable of converting carbon dioxide into oxygen via photosynthesis and have been proposed as significant players in the evolution of current atmospheric oxygen levels during the Precambrian era (Meeks et al. 2001; Schirrmeister et al. 2013) and as origin of chloroplasts via endosymbiosis with eukaryotic cells (Agapakis et al. 2011; Chu et al. 2004). The economic significance of cyanobacteria centers on their potential as synthesis platform for biofuels and other natural products (Machado and Atsumi 2012; Rosgaard et al. 2012); and gene clusters coding for novel enzymes that catalyze unique chemical reactions have been identified from several cyanobacterial genomes (Kleigrewe et al. 2016; Zarzycki et al. 2013). Access to cyanobacterial genomes with up-to-date annotation will provide an improved framework for studying and understanding the biology of this phylogenetic group that is essential for the global carbon and nitrogen cycle and to develop genetic tools that will facilitate the engineering of cyanobacteria for natural products synthesis. *Nostoc punctiforme* is a filamentous cyanobacterium found in the majority of illuminated environments and it plays a key role in the global nitrogen cycle through its facultative diazotrophic traits. Its life cycle is complex with vegetative cells differentiating into heterocysts, hormogonia and akinetes. The genome of *N. punctiforme* has been described previously as a large bacterial genome approaching 10 million (M) base pairs (bp) and the most recent genome sequence of the type strain *N. punctiforme* ATCC 29133 was released into the National Center for Biotechnology Information’s (NCBI’s) public nonredundant (nr) database more than 15 years ago (Meeks et al. 2001). Since then, this article has been cited more than 170 times and the initial annotation of this genome has been extensively utilized to identify genes involved in the carbon and nitrogen fixation process mediated by *N. punctiforme* and related species. This highlights the importance of this microorganism as a model system and the value of an up-to-date annotation of its genome. The *N. punctiforme* ATCC 29133 genome that was available through NCBI’s nr database (GenBank ID: CP001037.1) prior to this study contained 9,059,191 bp with approximately three quarters (77.44%) of its genome sequence coding for genes. Of the 6690 protein-coding genes, 2601 (38.3%) had no functional annotation, which subsequently renders a comprehensive understanding of their physiological role and the metabolic capability of the complete genome rather challenging. The rapid improvements of sequencing technologies and sequence analysis tools in the past decade has extended our ability of generating and mining genomic information to several levels beyond what was possible more than a decade ago (Gomez-Escribano et al. 2016; Shendure and Lieberman Aiden 2012) when the first version of the *N. punctiforme* ATCC 29133 genome was released. For instance, sequencing of bacterial genomes using Pacific Biosciences’s (PacBio’s) single molecule sequencing approach that simultaneously allows the detection of deoxyribonucleic acid (DNA) methylation patterns in bacterial genomes is now routinely performed in many laboratories (Flusberg et al. 2010; Gomez-Escribano et al. 2016; Koren et al. 2013). Moreover, the ample development of bioinformatics tools with various strategies for genome assembly and annotation has dramatically increased our ability to understand the biology of prokaryotes and eukaryotes at the genomic level and to mine their genomes for natural products (Weber et al. 2015; Medema et al. 2015; Yandell and Ence 2012). In this context, the objective of this study is to provide an update of the *N. punctiforme* genome through resequencing and reannotating of its genome with state-of-the-art technologies. The data presented here were generated and analyzed as part of Community Science Program (Project ID 1393) at the Department of Energy’s Joint Genome Institute (DOE’s JGI) and the sequenced and annotated genome are publically available through the Joint Genome Institute’s Integrated Microbial Genomes and Microbiomes (IMG/M; https://img.jgi.doe.gov/cgi-bin/mer/main.cgi) system (Markowitz et al. 2012) samples warehouse under the IMG Submission Identifier (ID) 62757. The data can be explored and analyzed using the IMG/M system or downloaded for further analyses using stand-alone tools.

*Nostoc punctiforme* ATCC 29133 is an aerobic, Gram-negative, filamentous and motile cyanobacterium with photosynthetic and nitrogen-fixing capabilities. *Nostoc punctiforme* is found in illuminated terrestrial environments and has a complex life cycle with vegetative cells differentiating into heterocysts, hormogonia and akinets. Vegetative cells are often larger than hormogonium cells with a diameter between 5 and 6 mm. The hormogonium cells institute infection for the symbiosis between cyanoba cterium and plants and are 1.5–2 mm in diameter (Meeks et al. 2001; Meeks and Elhai 2002). The heterocysts are 6–10 mm in diameter whereas akinetes are 10–20 mm. The functioning of oxygen sensitive nitrogenases is ensured by the heterocyst’s glycolipid layer, which provides a barrier for gases. Moreover, the differentiation of vegetative cells into the spore-like akinetes is determined by environmental factors for example through light limitation (Adams and Duggan 1999). Besides its photoautotrophic mode, *N. punctiforme* growth has been reported in the absence of light but with availability of sucrose, glucose or fructose (Meeks et al. 2001). General features and information about *N. punctiforme* ATCC 29133 are summarized in Table 1.

**Table 1:** Classification and general features of *Nostoc punctiforme* ATCC 29133

Property	Term	Evidence code^a^
Classification	Domain *Bacteria*	TAS (Woese et al. 1990)
	Phylum *Cyanobacteria*	TAS (Castenholz 2015)
	Class *Cyanophyceae*	
	Order *Nostocales* (Subsection IV)	TAS (Rippka et al. 2015)
	Family *Nostocaceae* (Subsection IV.I)	TAS (Whitman 2015)
	Genus *Nostoc*	TAS (Herdman et al. 2015)
	Species *punctiforme*	TAS (Herdman et al. 2015)
	Strain ATCC 29133/PCC 73102	
Gram stain	Negative	TAS (Hoiczyk and Hansel 2000)
Cell shape	Filamentous	TAS (Herdman et al. 2015)
Motility	Motile	TAS (Lehner et al. 2011)
Growth temperature	26 °C	IDA
pH	7.1	IDA
Habitat	Fresh water, Soil	TAS (Herdman et al. 2015)
Oxygen requirement	Aerobic	TAS (Herdman et al. 2015)
Biotic relationship	Symbiotic	TAS (Herdman et al. 2015)
Pathogenicity	Non-pathogen	NAS
Geographic location	USA/Washington	
Sample collection	October 10th 2014	
Latitude	46.3119	
Longitude	−119.263	

^a^Evidence codes—IDA: Inferred from direct assay; TAS: traceable author statement (i.e., a direct report exists in the literature); NAS: non-traceable author statement (i.e., not directly observed for the living, isolated sample, but based on a generally accepted property for the species, or anecdotal evidence). Evidence codes are from the Gene Ontology project (Ashburner et al. 2000)

We selected *N. punctiforme* ATCC 29133 for genome resequencing and reannotation due to its importance in the global carbon and nitrogen cycle, its potential biotechnological applications and the value and impact of the first publicly available version of its genome on the scientific community. It is very likely that an updated annotation of the *N. punctiforme* ATTC 29133 genome will benefit future genome explorations that target genes associated with carbon and nitrogen fixation and the production of biofuels and value-added chemicals.

## Materials and methods

### Growth conditions and genomic DNA preparation

*Nostoc punctiforme* ATCC 29133 was obtained from the American Type Culture Collection (ATCC) and grown at 26 °C in a 75 cm^2^ corning vent cap tissue flask using 30 mL of ATCC’s 616 medium under diurnal conditions with 16 h of light exposure followed by 8 h of darkness. After 12 days, 10 mL of cell culture were concentrated by centrifugation at 10,000 rpm for 10 min. Cells were resuspended in 1 mL fresh media and used for DNA extraction. DNA was extracted using MP Biomedicals’ FastDNA™ SPIN Kit for Soil DNA extraction kit according to the manufacturer’s protocol. DNA was suspended in 100 µL DNase/pyrogen-free H_2_O and DNA concentration was determined using a Qubit 2.0 fluorometer (Life Technologies, Grand Island, NY) according to the manufacturer’s protocol.

### Genome sequencing and assembly

The draft genome of *N. punctiforme* ATCC 29133 was generated at DOE’s JGI using PacBio’s single molecule real-time sequencing technology (Eid et al. 2009). A > 10 kbp PacBio SMRTbell™ library was constructed and sequenced on the PacBio RS platform, which generated 446,884 filtered subreads totaling 915.4 Mbp. A description of the library construction and sequencing performed at the JGI can be found at http://www.jgi.doe.gov. The raw reads were assembled using hierarchical genome-assembly process (HGAP version: 2.3.0, protocol version = 2.3.0 method = RS HGAP Assembly.3, smrtpipe.py v1.87.139483) (Chin et al. 2013). The final draft assembly contained 65 contigs in 65 scaffolds, totaling 9.064 Mbp in size. The obtained read coverage was 96.8-fold. Sequencing and assembly statistics are summarized in Tables 2 and 3.

**Table 2:** Sequencing and assembly information

MIGS ID^a^	Property	Term
MIGS 31	Finishing quality	High quality draft
MIGS-28	Libraries used	>10 kbp PacBio SMRTbell
MIGS 29	Sequencing platform	PacBio SMRT RS
MIGS 31.2	Fold coverage	96.8-fold
MIGS 30	Assembler	HGAP 2.3.0

^a^Field et al. 2008

**Table 3:** Genome statistics for *Nostoc punctiforme* ATCC 29133

Attribute	Meeks et al. (2001)		This study	
	Value	% of total	Value	% of total
Genome size (bp)	9059191	100	9064343	100
DNA coding (bp)	7015747	77.44	7393120	81.56
DNA G+C (bp)	3746385	41.35	3751137	41.38
DNA scaffolds	6	100	65	100
Total genes	6791	100	7775	100
Protein coding genes	6690	98.51	7664	98.57
RNA genes	101	1.49	111	1.43
Genes with function prediction	4089	60.21	5076	65.29
Genes without assigned function	2601	38.3	2588	33.29
Genes assigned to COGs	3432	50.54	3598	46.28
Genes with Pfam domains	5010	73.77	5381	69.21
Genes with signal peptides	274	4.03	297	3.82
Genes with transmembrane helices	1525	22.46	1635	21.03
Genes in biosynthetic clusters	602	8.86	1080	13.89
CRISPR repeats	8		10	

### Genome annotation

Genes were identified using Prodigal (Hyatt et al. 2010) and the predicted coding DNA sequences (CDSs) were translated and used to search the NCBI nr database, as well as the UniProt, TIGRFam, Pfam, KEGG, COG, and InterPro databases. The tRNAScanSE tool (Lowe and Eddy 1997) was used to find transfer ribonucleic acid (tRNA) genes, whereas ribosomal RNA (rRNA) genes were found by searches against models of the rRNA genes built from SILVA (Pruesse et al. 2007). Other non-coding RNAs such as the RNA components of the protein secretion complex and the RNase P were identified by searching the genome for the corresponding Rfam profiles using INFERNAL (Nawrocki et al. 2009). Additional gene prediction analysis and manual functional annotation was performed within the JGI’s Integrated Microbial Genome platform (Markowitz et al. 2010).

## Results

### Genome properties

The genome of *N. punctiforme* ATCC 29133 generated in this study is 9,064,343 bp in length with 7,393,120 (81.56%) coding base pairs and a GC content of 41.38%. Assembly of the sequences produced 65 DNA scaffolds of 65 contigs and a total of 7775 genes for which 7664 (98.57%) were identified as protein coding genes and 111 (1.43%) were identified as RNA genes. In addition, 5076 genes (65.29%) had a predicted function, which increases the number of *N. punctiforme* ATCC 29133 genes with functional annotation by ~24%. Of the identified genes, 3598 (46.28%) were assigned to Clusters of Orthologous Groups (COGs) categories. Genome statistics and distribution of genes into COGs functional categories are presented in Tables 3 and 4 respectively. In short, the COGs functional category with the largest number of assigned genes was identified as signal transduction mechanisms with 313 genes (7.71%), followed by the cell wall/membrane biogenesis functions with 277 genes (6.83%). Furthermore, 564 genes (13.9%) had a “general function” prediction, 270 genes with unknown function (6.65%) and 4177 genes (53.72%) were not assigned to any COG category.

**Table 4:** Number of genes associated with general COG functional categories

Code	Meeks et al. (2001)		This study		Description
	Count	%^a^	Count	%^a^	
E	246	6.23	245	6.04	Amino acid transport and metabolism
G	185	4.69	192	4.73	Carbohydrate transport and metabolism
D	43	1.09	39	0.96	Cell cycle control, cell division, chromosome partitioning
N	63	1.6	67	1.65	Cell motility
M	276	6.99	277	6.83	Cell wall/membrane/envelope biogenesis
B	2	0.05	2	0.05	Chromatin structure and dynamics
H	240	6.08	240	5.91	Coenzyme transport and metabolism
V	144	3.65	153	3.77	Defense mechanisms
C	207	5.24	210	5.17	Energy production and conversion
W	20	0.51	23	0.57	Extracellular structures
S	242	6.13	270	6.65	Function unknown
R	553	14.01	564	13.9	General function prediction only
P	233	5.9	237	5.84	Inorganic ion transport and metabolism
U	37	0.94	38	0.94	Intracellular trafficking, secretion, and vesicular transport
I	143	3.62	144	3.55	Lipid transport and metabolism
–	43	1.09	68	1.68	Mobilome: prophages, transposons
F	76	1.93	74	1.82	Nucleotide transport and metabolism
O	198	5.02	204	5.03	Posttranslational modification, protein turnover, chaperones
A	1	0.03	1	0.02	RNA processing and modification
L	138	3.5	140	3.45	Replication, recombination and repair
Q	180	4.56	194	4.78	Secondary metabolites biosynthesis, transport and catabolism
T	318	8.05	313	7.71	Signal transduction mechanisms
K	155	3.93	159	3.92	Transcription
J	205	5.19	204	5.03	Translation, ribosomal structure and biogenesis
–	3359	49.46	4177	53.72	Not in COG

^a^Based on the total number of protein coding genes

**Fig. 1:**
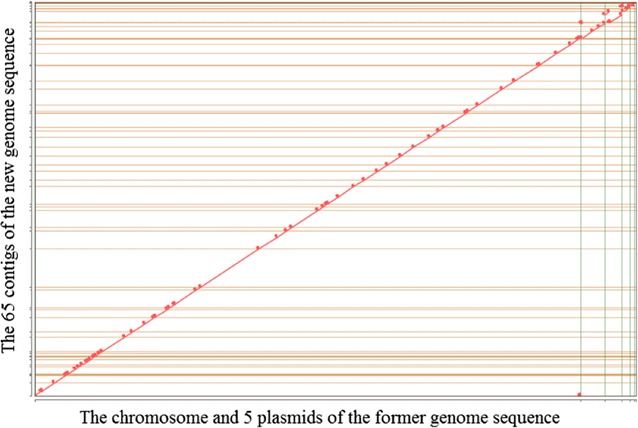
Alignment plot of *Nostoc punctiforme* ATCC 29133 genomes

To evaluate how the assembled genome sequence from this project compares to the *N. punctiforme* genome released by the JGI in 2014 (GenBank ID: CP001037.1; not published), we aligned both assembled genomes to each other. Alignment was performed using MUMmer 3.0 (Kurtz et al. 2004) with the previously released genome sequence as the reference. The genomic sequence similarity plot (Fig. 1) suggests high agreement between the genome sequences. The JGI IMG portal genome comparison tools indicate an average nucleotide identity of 99.99%, a fraction of orthologous genomic regions of 0.98 and 0.92 and 6606 bidirectional best hits. In contrast, the genome presented here consists of 9064343 bp compared to the previously reported 9059191 bp, which might be due to the larger amount of sequence generated, but it is also possible that this is caused by the improved assembly algorithms that was developed since the first genome assembly was performed and that were employed for our data analysis. Likewise, we have identified 7775 genes with 7664 genes identified as protein-coding genes and 5076 genes with function prediction. In the initial assembly performed by the JGI in collaboration with Meeks and colleagues only 6791 genes were identified, with 6690 of these genes identified as protein-coding genes and 4089 of these genes with a functional prediction. The initial assembly has also been uploaded and can be accessed and utilized for future analyses and studies through the JGI’s IMG system using the IMG Genome ID 642555144 (https://img.jgi.doe.gov/cgi-bin/m/main.cgi?section=TaxonDetail&page=tax onDetail&taxon_oid=642555144).

## Discussion

The advent of affordable second and third-generation DNA sequencing resulted in a significant reduction of costs per sequence supporting the democratization of genome sequencing (Caporaso et al. 2012). Sequencing microbial genomes is now a standard procedure performed in many laboratories and third-generation sequencing technologies, such as PacBio’s SMRT Sequencing platform, facilitate to generate almost complete high-quality genomes from archaea and bacteria in only a few hours (Eid et al. 2009; Rice et al. 2016). This development resulted in a vast amount of genomic information that is now available through public database such as NCBI’s GenBank (https://www.ncbi.nlm.nih.gov/genome/browse/) or JGI’s IMG (https://img.jgi.doe.gov/). These two databases alone contained a total of 89971 and 47009 prokaryotic genome submissions, including 5590 and 26707 non-redundant complete prokaryotic genomes, when accessed on December 27th 2016 respectively. With this almost infinite and continuously growing amount of sequence information, identification and extraction of relevant and accurate data becomes a major challenge. Microbiologists are nowadays limited mostly by the relatively low throughput of molecular experiments that remain necessary to test the functionality and biological role of proteins newly identified using in silico approaches. This renders accurate functional prediction as key to an enhanced understanding of biological processes (Jiang et al. 2016). The importance of an accurate function prediction is even more pronounced in organisms that function as model systems and whose genomes serve as reference for the analyses of phylogenetically related genomes (Cormier et al. 2016); which explains the growing interest in improving the genome annotation of model system microorganisms such as *Bacillus pumilus* (Gioia et al. 2007; Stepanov et al. 2016) and *Pichia pastoris* (*Komagataella phaffi*) (Valli et al. 2016; De Schutter et al. 2009), whose genomes were published during the early phase of the genomics era.

*Nostoc punctiforme* is an important model system for examining genomic and phenotypic properties of cyanobacteria and their biological carbon and nitrogen fixation capabilities (Sandh et al. 2014). An additional interest in the enhanced understanding of the *N. punctiforme* genome has been sparked by the increased utilization of cyanobacteria as synthesis platform of biofuels and other natural products (Machado and Atsumi 2012; Rosgaard et al. 2012). For these reasons we selected the type strain *N. punctiforme* ATCC 29133 for resequencing and reannotation with the assumption that an improved genome sequence and annotation would be of significant value to the scientific community. The newly sequenced, assembled and annotated *N. punctiforme* ATCC 29133 genome presented here, consists of 984 more genes and 987 more genes with predicted function compared to the previously available *N. punctiforme* ATCC 29133 genome. The improved gene calling and ability to predict gene function is most likely the result of combination of improved sequence quality and gene annotation algorithm that are now available. It can be anticipated that increasing the repertoire of genes with assigned function within the new *N. punctiforme* ATCC 29133 genome by ~24% might allow to fillin some of the knowledge-gaps that exist in our current understanding of the molecular processes during microbial carbon and nitrogen fixation. The new *N. punctiforme* genome also contained ~79% more biosynthetic clusters when compared to the previous version, opening new opportunities to explore the capability of *N. punctiforme* and its phylogenetically related neighbors for secondary metabolite synthesis.

Access to a reannotated *N. punctiforme* ATCC 29133 genome through a versatile genome browser that allows to explore DNA sequence data without any or only limited bioinformatics skills will enhance the ability of the scientific community to mine this genome for new insights into genes and gene pathways associated with carbon and nitrogen fixation and secondary metabolite synthesis. It might furthermore facilitate the identification of the genetic properties that might be responsible for some of the metabolic functions and phenotypes associated with *N. punctiforme* and close relatives. In the case presented here, we opted to load and provide the genome sequence and annotation through IMG/M, a system that has been maintained by the Department of Energy for over a decade and that facilitates comparative analysis and visualization of multilayered omics data (Chen et al. 2016). Subsequent analysis can be performed within the system using a variety of the visualization and analysis tools that have been implemented since the first release of this genome data analysis system in 2005 (Markowitz et al. 2008). Of particular interest for users might be the gene neighborhood function within the Biosynthetic Cluster option, through which the global structure as well as the nucleotide sequence of individual genes and gene clusters can be retrieved for subsequent wet-lab experiments. As new omics data, such as proteomic and transcriptomic data, become available, they can also be integrated easily into the IMG/M structure to complement the reannotated *N. punctiforme* ATCC 29133 genome data generated during this study. To increase the value of the genome data presented here, sequence data and annotation can also be downloaded through IMG/M and the JGI’s website and analyzed with other genome analysis software, including genome annotation pipelines and analysis systems such as SEED, RAST (Overbeek et al. 2014; Aziz et al. 2008) and the Department of Energy Systems Biology Knowledgebase (KBase; http://kbase.us).

To maximize the benefit of genomes generated with federal funding and to provide access to up-to-date sequence information without the need of in-depth bioinformatics skills, we advocate to resequence and annotate microbial type strains that were sequenced in the early stage of the genomics era, with up-to-date sequencing platforms and improved annotation software and to make these genomes and their annotations available to the scientific community through user-friendly genome browsers, such as the JGI’s IMG/M, at the time of publication.

## Abbreviations

M: million
bp: base pairs
nr: nonredundant
NCBI: National Center for Biotechnology Information
PacBio: Pacific Biosciences
DNA: deoxyribonucleic acid
DOE: Department of Energy
JGI: Joint Genome Institute
IMG/M: Integrated Microbial Genomes and Microbiomes
ID: identifier
ATCC: American Type Culture Collection
CDSs: coding deoxyribonucleic acid sequences
tRNA: transfer ribonucleic acid
rRNA: ribosomal ribonucleic acid
COGs: Clusters of Orthologous Groups
